# Acute Myocardial Infarction as Initial Manifestation of Acute Myeloid Leukemia: A Rare Manifestation of Leukostasis

**DOI:** 10.7759/cureus.9551

**Published:** 2020-08-04

**Authors:** Dharmini Manogna, Ronald Sham

**Affiliations:** 1 Internal Medicine, Rochester Regional Health, Rochester, USA; 2 Hematology/Oncology, Rochester Regional Health, Rochester, USA

**Keywords:** leukemia, myocardial infarction, leukostasis, acute leukemia, hyperleukocytosis, leukapheresis

## Abstract

Leukostasis is a medical emergency caused by compromise of tissue perfusion secondary to hyperleukocytosis in acute myeloid leukemia (AML). Typically it affects lungs and brain, with cardiac involvement being exceedingly rare. We present a case of AML presenting as acute coronary syndrome secondary to leukostasis-induced myocardial ischemia. A 43-year-old morbidly obese gentleman presented with typical anginal chest pain. On examination, he was diaphoretic and in acute distress secondary to pain. EKG revealed ST elevation in lead I and aVL and PR depressions in precordial leads. Troponin peaked at 5.55 ng/mL. Echocardiogram showed normal left ventricle function with no wall motion abnormality. Blood work was notable for white blood cell (WBC) count of 185,200 cells/μL with 81% blasts. Coronary angiogram revealed no obstruction. Emergent leukapheresis and hydroxyurea were initiated. WBC count decreased to 48,200 cells/ μL and angina resolved after leukapheresis. With diagnosis of AML, he received 7+3 induction chemotherapy with cytarabine and idarubicin, followed by re-induction and consolidation chemotherapy. He subsequently underwent allogenic bone marrow transplantation and achieved complete remission. Hyperleukocytosis in AML can cause leukostasis, characterized by evidence of tissue ischemia. Coronary vasculature accounts for 6% of cases with leukostasis. This can manifest as myocardial infarction. Emergent and timely initiation of leukapheresis can potentially lead to a complete resolution of microvascular occlusion.

## Introduction

Higher leukocyte count at initial presentation has been reported to be associated with increased morbidity and mortality in acute myeloid leukemia (AML) [1]. Leukemic blasts can have a significant effect on blood viscosity, which is particularly pronounced in the microvasculature [2]. Leukostasis is an acute medical emergency caused by compromise of tissue perfusion secondary to hyperleukocytosis. It most commonly occurs in AML. Typically it affects lungs and brain, with cardiac involvement being exceedingly rare. We would like to highlight our experience of a rare case of AML presenting as acute coronary syndrome secondary to leukostasis-induced microvascular myocardial ischemia.

## Case presentation

A 43-year-old man presented with abrupt onset of chest pain, which started while he was having dinner. The pain was severe, crushing pain on the left side of the chest. It radiated to left shoulder and aggravated with exertion. He reported diaphoresis and shortness of breath. He had noticed swelling of his gums over the preceding few weeks. There were no prior episodes of exertional chest pain. A carpenter by profession, he was not sedentary and reported no recent long-distance travel, immobilization or leg swelling. Clinical history was notable for absence of fever, night sweats, anorexia, weight loss or other constitutional symptoms. Pertinent past medical history included hypertension, hyperlipidemia, pre-diabetes and morbid obesity. He was on Lisinopril and statin and admitted to non-compliance. Family history was negative for cardiac diseases. The patient was a former smoker with 12 pack-years history of smoking. On examination, he was diaphoretic and in acute distress secondary to chest pain. Pulmonary and cardiac examinations were unremarkable. No jugular venous distension, hepatojugular reflex, organomegaly or peripheral edema were appreciated.

**Figure 1:**
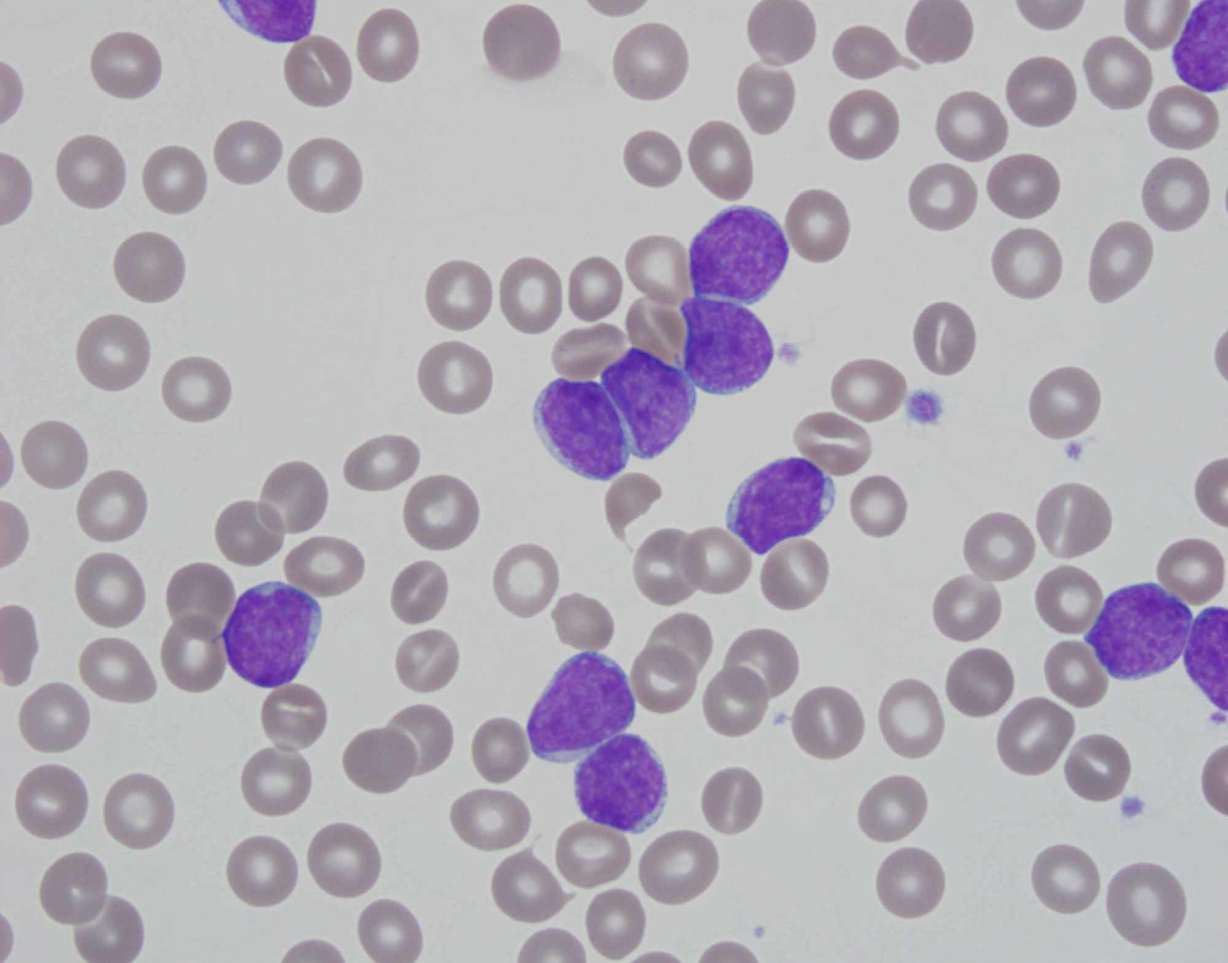
Peripheral blood smear Peripheral blood smear on admission revealing > 80% blast forms

**Figure 2:**
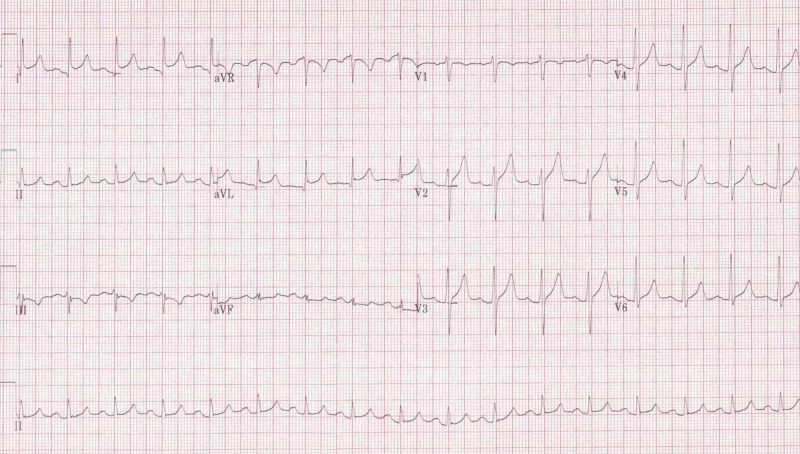
Admission EKG EKG on admission revealing elevation of ST segment in leads I and aVL, along with PR depression in the precordial leads

Complete blood count revealed leukocyte count of 185,200 cells/µL. Peripheral blood smear demonstrated 81% blasts (Figure 1). Lactate dehydrogenase was 315 U/L. EKG showed ST elevation in leads I and aVL along with PR depression in the precordial leads (Figure 2). Troponin-I peaked at 5.55 ng/mL. Coronary angiogram was unremarkable. Circulating blasts were consistent with AML. On further workup, he was diagnosed with FMS-like tyrosine kinase-3 (FLT-3) negative, nucleophosmin-1 (NPM-1) negative, isocitrate dehydrogenase 1-2 (IDH 1-2) negative AML. Chromosomal analysis noted 46,XY,add(2)(p11.2),-12,add(12)(p13),+mar(9)/46,XY(11) abnormality. AML fluorescence in situ hybridization panel was normal. Next-generation sequencing studies revealed a BCL-6 corepressor (BCOR) mutation.

**Figure 3:**
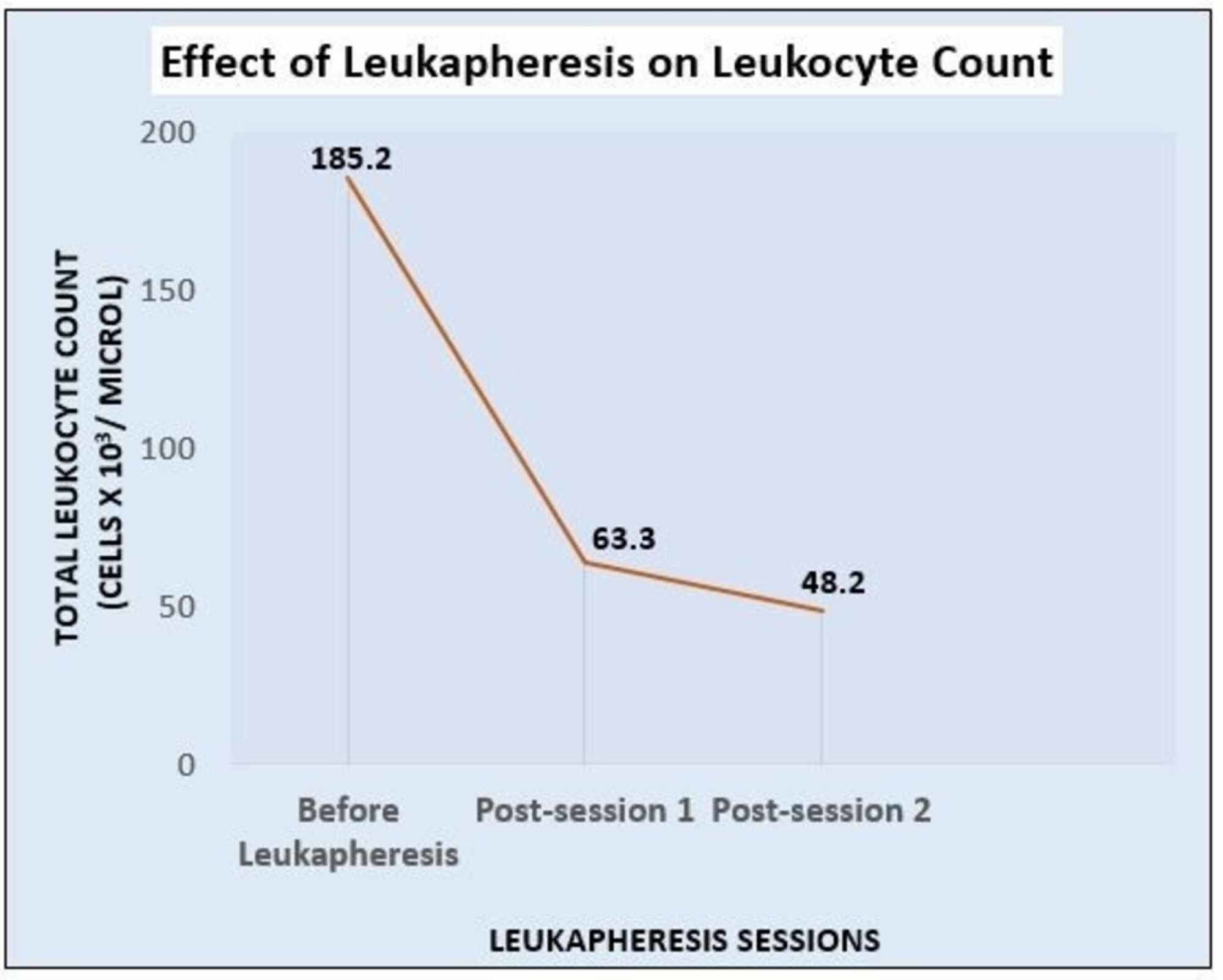
Effect of leukapheresis on leukocyte count Graphical representation of decrease in leukocyte count after two sessions of leukapheresis

Nitroglycerine infusion was started for ST elevation myocardial infarction (MI). Hydroxyurea and leukapheresis were initiated on an emergent basis, following which the leukocyte count decreased to 48,200 cells/µL (Figure 3). There were initial concerns regarding possible myopericarditis and thus, colchicine and aspirin were added. They were subsequently discontinued as chest pain resolved during the first session of leukapheresis.

Induction chemotherapy with cytarabine and idarubicin was initiated. Patient received re-induction therapy with fludarabine/cytarabine and granulocyte colony-stimulating factor (FLAG regimen) due to persistent disease, followed by two cycles of consolidation chemotherapy with high dose cytarabine. With subsequent hematopoietic stem cell transplantation, he achieved complete remission.

## Discussion

The initial presenting features of AML are secondary to the underlying cytopenias, which manifest as fatigue, infections and hemorrhagic symptoms. An increased burden of circulating leukemic cells can cause hyperleukocytosis, which has been defined variably as a WBC count of greater than 50,000 or 100,000 cells/mL. Leukostasis is characterized by the presence of symptoms of end-organ ischemia. Hyperleukocytosis, by itself, has been noted to be a poor prognostic factor in AML as it can predispose to potentially fatal complications such as disseminated intravascular coagulation, tumor lysis syndrome and leukostasis [1].

Leukostasis results in increased blood viscosity which can lead to ischemic and thrombotic complications. As reported by Litchman and Rowe, the mean cell volume of leukemic myeloblasts ranges from 350-450 cubic microns, compared to leukemic lymphoblasts which range from 250-350 cubic microns [2]. Further, myeloblasts are much less deformable. Therefore, leukostasis occurs most commonly in AML and chronic myeloid leukemia, and is rarely seen in chronic lymphocytic leukemia even with higher WBC counts. It typically affects the microvasculature of cerebral, pulmonary and renal circulations. Cardiac microvascular occlusion causing myocardial infarction has seldom been described as the sole presenting symptom of AML. A retrospective study done by Stahl et al that analyzed patients with newly diagnosed AML with leukocyte count > 50,000 cells/ µL studied organs affected by leukostasis and found that lungs and CNS are most frequently affected with the incidence of 44% and 36%, respectively. On the contrary, cardiac involvement accounted for 6% of cases [3]. Besides myocardial infarction, there have been case reports of leukocytosis causing cardiac failure as well [4,5].

There have only been a handful of cases that have reported MI as the initial presentation of acute leukemia [6-8]. Majority of these cases were described in the setting of disseminated intravascular coagulation (DIC) in patients with acute promyelocytic leukemia. Acute coronary syndrome in other types of AML is exceedingly rare.

Leukapheresis involves the withdrawal of whole blood, separation of leukocytes and re-infusion of the remainder into the patient. It is typically reserved for symptomatic patients with hyperleukocytosis, until it is safe to administer induction chemotherapy. Earlier studies have shown variable results with leukapheresis. Choi et al studied the effect of therapeutic leukapheresis on early complications and outcomes in patients with AML and hyperleukocytosis. Their results did not demonstrate any effect on survival outcome and incidence of early complications including tumor lysis syndrome and DIC [9]. However, it may be noted that only leukocyte count was taken into consideration, irrespective of the presence of leukostatic symptoms. Villgran et al. suggested that initial leukapheresis may prevent complications of hyperleukocytosis in a subgroup of patients who present with hyperleukocytosis until confirmation of AML diagnosis and initiation of systemic therapy [10].

Our patient's presentation was unique - the sole symptom was ischemic chest pain. He had hyperleukocytosis on presentation, complicated by leukostasis manifesting as acute ST elevation MI. This is unusual given that the CNS and lung have been classically known to be affected by leukostasis. Our patient had several cardiac risk factors and likely had background inflammation of his cardiac vasculature, which may have predisposed these vessels to be affected.

## Conclusions

Acute coronary syndrome can be a rare presentation of AML. Leukostasis is an acute hematological emergency caused by compromise of tissue perfusion secondary to a marked elevation of leukocyte count. Cardiac involvement is uncommon. Coronary angiogram may be unrevealing. Timely initiation of leukapheresis can potentially lead to complete resolution of microvascular occlusion. Our experience with this patient highlights the importance of recognition of an uncommon presentation of acute leukemia.
